# Real-world examination of the rates of long-acting injectable attrition in a cohort of early psychosis patients after discharge from an early intervention service for psychosis

**DOI:** 10.1177/20451253251384509

**Published:** 2025-10-16

**Authors:** Candice E. Crocker, Thomas Hastings, Marc-Andre Roy, Nicola Otter, Philip G. Tibbo

**Affiliations:** Department of Psychiatry, Dalhousie University, NSEPP, Rm 3030, Abbie J. Lane Bldg, 5909 Veteran’s Memorial Lane, Halifax, NS B3H 2E2, Canada; Department of Diagnostic Radiology, Dalhousie University, Halifax, NS, Canada; Department of Psychiatry and Behavioural Neuroscience, McMaster University, Oakville, ON, Canada; Department of Psychiatry and Neurosciences, Faculté de Médecine de l’Université Laval, Quebec, QC, Canada; Canadian Consortium for Early Intervention in Psychosis, Hamilton, ON, Canada; Department of Psychiatry, Dalhousie University, Halifax, NS, Canada

**Keywords:** antipsychotic medication, early intervention, long acting injectables, long-term follow up

## Abstract

**Background:**

Patients treated in early intervention for psychosis programs have better treatment outcomes and higher rates of long-acting injection (LAI) antipsychotic medication utilization (20%–50%) versus treatment as usual. These programs usually serve patients for 2–3 years, then most patients are discharged to other mental health services and studies of patients with longer-standing schizophrenia suggest switching to oral medications may be common. However, following patients post-discharge is complicated by the challenges of migrated patient records across clinical services and providers.

**Objectives:**

To examine whether LAI use continues after discharge from an early intervention service for psychosis.

**Design:**

This study was a retrospective cohort study examining the effects of continuation or discontinuation of LAI therapy in individuals who have completed treatment in an early intervention service (EIS) for psychosis.

**Methods:**

A retrospective cohort was created from a group of individuals discharged from EIS for psychosis over a 3-year period from January 1, 2016 to December 31, 2018 and followed for mental health outcomes and antipsychotic medications prescribed for a subsequent 2-year period at discharge, 6, 12, 18, and 24 months post-discharge.

**Results:**

Of 85 subjects discharged from three sites in three different provinces in Canada for whom full follow-up could be recorded, 60 subjects remained on LAI medications after 24 months (71%). The average age of the cohort was 22 years (SD 4.7) at admission to an EIS. At discharge, the most commonly used LAI was aripiprazole, and most subjects were maintained on the same formulation at 24 months, if still on LAI. Reasons for discontinuation were predominantly patient preference. Significant differences in clinical outcomes, measured through reduced rehospitalization were seen for those who remained on LAI as compared to those who did not.

**Conclusion:**

LAI adherence is still strong 24 months after discharge from an EIS for psychosis.

## Plain language summary

Examination of the use of long acting injectable (LAI) antipsychotic medications in early psychosis patients after discharge from an early intervention service for psychosis

Why was this study done? Patients who are treated in early intervention medical services for psychosis (EIS) have better treatment outcomes. Patients in these programs also have higher rates of long-acting injection antipsychotic (LAI) medication prescriptions (20-50%) compared to treatment by regular psychiatrists. EIS usually serve patients for 2-3 years, and then most patients are discharged to other mental health services. After moving to a new treatment program, some studies have shown that patients with chronic illness switch to oral/pill medications instead of injections. However, following mental health patients over time is complicated by the challenges of following patient records across different hospitals and doctors. How was the study done? We set out to collect data on a group of patients that had left an early intervention service and were using LAI medications. We followed their care for two years after discharge to see if these patients stayed on LAIs with their new doctors. What did the study find? We found that of 85 subjects discharged from 3 sites in 3 different provinces in Canada for whom all medical records could be found, 60 patients remained on LAI medications after 24 months (71%). The average age of the patients was 22 years (SD 4.7) at admission to an EIS. At discharge the most commonly used LAI was aripiprazole and most patients stayed on this same drug for the full 24 months. We recorded reasons for stopping LAIs and found that stopping was usually from the patient’s request. We found a significant benefit through reduced rehospitalization for were seen for those who remained on LAI as compared to those who did not. What does this mean? The take away message from this study is that LAI use is still strong 24 months after discharge from an EIS for psychosis.

## Introduction

Early intervention services (EIS) for psychosis provide integrated clinical and psychosocial services to individuals in the early phase of a psychotic disorder. Current guidelines outline best practices for EIS, including pharmacological and non-pharmacological treatment strategies.^1–4^ The use of long-acting injectable antipsychotic medication in EIS for psychosis has had a recent focus, due in part to the development of the second-generation antipsychotic long-acting injectable (LAI) formulations. Several meta-analyses, in individuals with more long-standing illness as well as those in the early phase of psychosis, have reported the benefits of LAIs on a number of different health outcomes, including health care utilization.^5–7^ In Canada, recommendations for the use of LAIs was published over a decade ago, and a recent cross-sectional survey of EIS for psychosis programs in Canada reported that 35.1% of their patients were on a LAI.^8,9^ LAI use in Canadian EIS for psychosis services was 24.7% in 2016 and 35.1% in 2020.^8^ In addition, the rate of prescribing of LAIs remained stable in Canada during the COVID-19 pandemic, suggesting many providers perceive continuity of LAI treatment as important.^10^

Studies comparing second-generation LAIs to similar oral antipsychotics have reported that persistence with medication is better with LAI use.^11^ However, there are other studies that contend that the dropout rate is high enough to make second generation antipsychotics (SGA)-LAI too expensive as a successful treatment regime, and alternatives should be considered.^12^ A more recent economic analysis showed that treatment with LAIs was associated with significantly reduced healthcare costs in 18–35 year olds with schizophrenia.^13^ There still remains a lack of studies investigating the rates of longer-term use of LAIs and their impact on treatment in the “real world,”^14^ specifically in the post-EIS setting.

Discharge to a new service is an important consideration for study, as changes in therapeutic treatment can impact a patient’s disease trajectory. The negative impacts of these changes are shown by evidence from several countries in a meta-analysis, suggesting that the benefits obtained in early psychosis programs are lost over time.^15^ In particular two randomized control trials (the Danish OPUS trial^16^ and the Lambeth Early Onset (LEO) from the United Kingdom^17,18^) showed that in individuals randomized to treatment as usual or an EIS for 2 years; at the 5 year follow-up time point, the treatment gains for those enrolled in the EIS arm (measured as reduced symptoms, reduced substance use, improved global functioning, increased treatment adherence and decreased hospitalization rates) are no longer significantly different from treatment as usual.^16,18,19^ These findings may be related to a number of factors; however, one that has not been investigated is the continuation/discontinuation of LAIs post-discharge from EIS. In an individual who was on an LAI, discontinuation may influence the potential benefits reported with LAI use on health services outcomes, as well as mitigating substance use effects on relapse is symptoms.^20^

This study examines the LAI use of a real-world cohort, examining the attrition rates from LAIs in patients transitioning from three early intervention programs for psychosis in Canada. We hypothesize that any deterioration in patient mental health may be related to attrition from LAIs in those discharged from EIS for psychosis on LAIs. We determined the 6, 12, 18, and 24-month rates of LAI use, as well as emergency department visits and hospitalizations for mental health-related issues for patients discharged over a 3-year period, with an exploratory analysis to examine the reasons for discontinuation in patients and whether these reasons were patient or provider driven.

## Methods

### Study population

This cohort study examined the rates and effects of continuation or discontinuation of LAI therapy in individuals who have completed treatment in an EIS for psychosis. Three Canadian sites participated, which were members of the Canadian Consortium for Early Intervention in Psychosis (www.epicanada.org). A retrospective cohort was created by first identifying every patient with a diagnosis of a primary psychotic disorder who was discharged from one of these EIS programs between January 1, 2016 and December 31, 2018. Exclusion criteria were patient was not on an LAI at the time of discharge. Then the cohort used for this study was the subset of all discharged patients who were receiving LAIs at the time of discharge. One hundred and twenty-eight patients met all three inclusion criteria (primary psychotic disorder, discharged during the study time period and stable on an LAI). Stable on an LAI was defined as on an LAI for 3 months prior to discharge with no medication changes. Medical records for these individuals for the 24-month period post-discharge were examined. The sites involved in this study were two, primarily English speaking and one primarily French speaking, and each was located in a different Canadian province, and thus, the results could be considered more generalizable. The STROBE guidelines for cohort studies were used to frame this report (www.strobe-statement.org).

### Demographic variables collected

Data variables were collected from the electronic medical record for each unique patient. Baseline variables were collected on age at clinic entry, age at clinic discharge, sex and gender (where available), as well as broad race and ethnicity data based on Statistics Canada Categories short list from 2021 Census.^21^

### Treatment measures

Several treatment variables were collected. Baseline treatment characteristics included the LAI treatment at time of discharge, including both medication name and dose, diagnosis at discharge if available and whether on a community treatment order (CTO). CTOs are a type of judicial involuntary treatment plan put in place while residing in the community that is permitted for use in Canada under a narrow set of guidelines.^22^ Whether the patient was on any LAI at 6, 12, 18 and 24 months post-EIS discharge was determined, as well as if LAI (medication/dose) use post-discharge matched what was used at the time of discharge. The status of LAI use at each time point was recorded as a dichotomous variable (on LAI/discontinued). A standardized measure of Clinical Global Impression (CGI-S) scores^23^ were used to assess overall clinical status. The stability of an individual’s mental health over the course of treatment from discharge from an early psychosis service until 24 months of follow-up by another service was also recorded as CGI-S scores to enable quantitative comparisons across sites.

Emergency department visits for mental health-related complaints and any records of hospitalizations (by number and duration of stay in days) for the four study time points (6, 12, 18, and 24 months post-discharge) were collected as another objective measure of an individual’s mental health status post-discharge. Any admission that bridged the time between intervals was entered as an admission in both time periods. For those who discontinued use over the 24-month follow-up time frame, the medical record was searched to try to determine if the discontinuation was driven by a formal request or refusal by the patient or if, in collaboration, the patient’s provider determined that the efficacy was lacking or the side effect profile was considered too therapeutically significant to continue use.

### Sample size calculation

Prior to project commencement, a power analysis was conducted. A power analysis was conducted based on the hypothesis that discontinuation after discharge would be similar to that observed in naturalistic observational studies looking from initiation of treatment to 12 months later. These studies tend to show rates of LAI discontinuation of between 28.8% and 39%.^24,25^ When we ran a power analysis using the more conservative 30% as the hypothesized proportion of discontinuations in a survival analysis, this gave a required sample size of 85 (with continuity correction) (https://sample-size.net/sample-size-survival-analysis/). The anticipated sample size at the time of study design was 200 individuals, and this was sufficient to test the hypothesis for the project.

### Data analysis

The percentage of patients still on antipsychotic LAI at 6, 12, 18 and 24 months was determined for each time point. Retention on an LAI, defined as consecutive time on treatment, was analyzed by a survival analysis using a Kaplan–Meier product limit estimator. The data were analyzed for potential differences in age, gender, diagnosis and presence of a CTO for those who stayed on LAIs at 24 months as compared to those who did not. Normality testing was conducted to ensure the underlying principles were not violated for the longitudinal data analysis.

Variables with significance between groups were used as covariates in the linear regression model to see if they played a role in defining the populations who maintained their LAI treatment compared to those who discontinued. Reasons for any LAI discontinuation were dichotomized into patient-driven and provider-driven reasons. The number of hospitalizations and ED visits related to mental health issues was recorded for each individual at each interval and over the entire 24-month period, with comparisons between those who continued versus those who discontinued LAI populations tested using a repeated measures analysis of variance.

## Results

### Patient demographics

The EIS programs that contributed data to this study were 2-, 3-, and 5-year programs, respectively, and as a result, both age at admission and age at discharge were collected. A total of 128 subjects fit the criteria for being discharged on an LAI during the period under study; however, the final cohort examined was 85 patients for whom 24 months of follow-up treatment data were available. The patients were predominantly male and identified as men. The LAIs reported in the final study cohort (broadly without regard to 1-month or 3-month formulations) were 53% on aripiprazole (*N* = 46), 39.5% on paliperidone (*N* = 34), and 5.8% fluanxol depot (*N* = 5). Twenty (23.5%) of the patients in the cohort were on a CTO at discharge from EIS. The demographic variables for the study participants are shown in Table 1.

**Table 1.** Demographic variables of the cohort.

Variable	Value	*N*
Age at admission (Avg + SD)	22.7 + 4.1	85
Biological sex (M/F)	77/8	85
Gender^a^	Man—94%	60
	Woman—6%	4
	Transgender/nonbinary/other not listed—0%	0
	Prefer not to answer—0%	0
Racial origin	North American origins—3%	2
	European origins—52%	41
	Caribbean origins—1%	1
	Latin, Central and South American origins—3%	2
	African origins—9%	7
	Asian origins—4%	3
	Oceanian origins—0%	0
	Other ethnic and cultural origins—4%	3
	Not mentioned-Not specified—25%	20
Diagnosis at discharge	Schizophrenia—71%	60
	Schizoaffective disorder—13%	11
	Unspecified schizophrenia spectrum disorder—12%	10
	Delusional disorder—2%	2
	Bipolar Type 1—2%	2

a Gender information was not available for all subjects.

### Rates of LAI use at 6, 12, and 18 months post-EIS discharge

**Figure 1.**
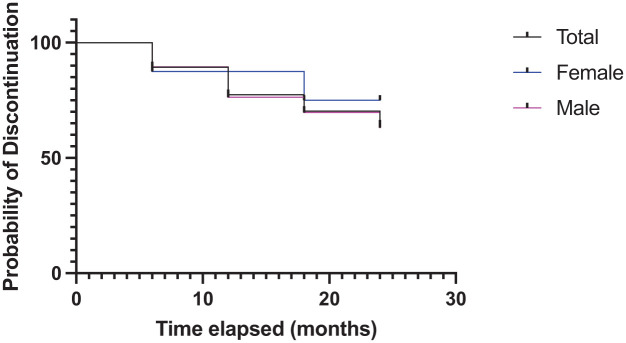
Kaplan–Meier analysis of the time to discontinuation for those who opted to stop LAI use. There was no association of sex, gender, LAI type or CTO with the choice to discontinue LAI medication. CTO, community treatment order; LAI, long-acting injectable.

The trajectories of LAI discontinuation were considered by Kaplan–Meier survival analysis, and there was no difference between males and females in the profiles of discontinuation (Figure 1). At 6 months post-discharge, nine patients had discontinued LAI use (10.5% discontinued). Discontinuation increased to 17 patients (20%) by 12 months and 23 patients (26%) by 18 months. At 24 months, 25 patients were not using (29.4%). However, while increasing in value, these results were not cumulative as individuals reported as discontinued in any epoch were not consistently the same patients. A total of five patients discontinued between 6 and 18 months, but had returned to LAIs by 24 months, for a total of 30 patients who discontinued during the course of the study. The timing of the 5 of 30 (16.6%) who switched back to LAI use by 24 months discontinued was two subjects at 6 months, two subjects at 12 months and a further one subject at 18 months. Four of these patients returned to the same LAI that they were on previously. The other individual discontinued LAI use, then returned to LAI use, but on a different LAI.

The profile differed somewhat in which LAIs were discontinued. A total of 54% of the cohort was on aripiprazole, and 33% of these individuals discontinued. A total of 32% of the cohort was on paliperidone 1-month formulation, and 18.5% discontinued, while 8% were on the 3-month formulation and 12.5% discontinued. The remaining 6% were on first-generation LAI medications, and 20% of that group discontinued use. However, there was no statistical impact of the choice of LAI on time to discontinuation.

### Reasons for discontinuation

Our second objective was to try to determine the reasons for discontinuation and whether the decision to stop LAI treatment was driven by patient choice, provider direction or through a substitute decision maker (SDM; in the case of a patient on a CTO). The medical record was searched for any indication of reasons for LAI discontinuation, and we found stated reasons for 24 of the 30 patient discontinuations (80% of the sample of individuals who discontinued). At a high level of categorization, 7 were provider initiated, 15 were patient initiated, and the remaining two were decisions made in consultation with substitute decision makers. Providers’ reasons were due to either side effect profiles or a lack of satisfactory response. Five of the provider switch choices were to use clozapine. The two SDM reasons were based on associated patients having significant side effect profiles that were affecting quality of life. Patient-driven reasons were less clear; however, overall, the reasons were most often “patient refused” (11 of 15). The other four changes were patients initiating a discussion of switching from the LAI to an oral formulation, with a stated aim to try to mitigate side effects or improve treatment response. A total of 7 of the 30 patients who discontinued were on a CTO at discharge from the EIS, and in each of these cases, the patient refused to continue with the LAI when the CTO expired.

### Association of use of LAIs with symptomatic stability

**Figure 2.**
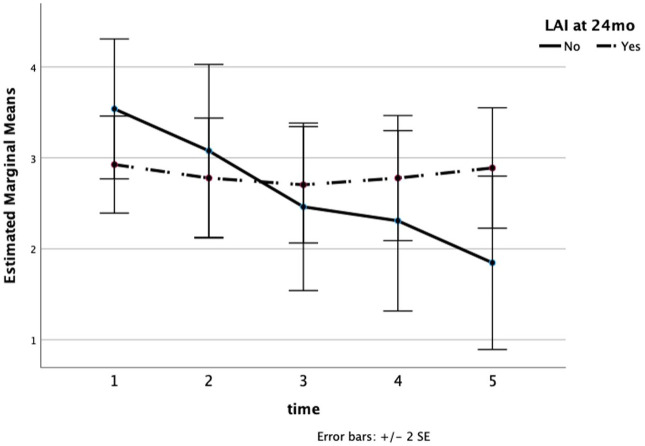
Estimated marginal means from modeling the relationship between LAI use at 24 months and CGI scores showed no difference between remaining on or discontinuing use of LAIs on symptom scores. CGI, Clinical Global Impression; LAI, long-acting injectable.

There was no association found between LAI use or discontinuation with the number of ED visits overall (F_1,83_ = 1.042, ns). The total number of ED visits across the cohort was low, with 7 ED visits between discharge to 6 months, 19 visits between 6 months and 12 months, 8 visits between 12 and 18 months and 23 visits between 18 and 24 months. However, total visits did not differ between groups based on LAI use. A repeated measures ANOVA was conducted to examine the effect of LAI use on inpatient psychiatric admissions at each timepoint. The number of hospitalizations at 14 total admissions between discharge to 6 months, 14 admissions between 6 months and 12 months, 7 admissions between 12 and 18 months, and finally 20 total admissions between 18 and 24 months. The main effect of LAI was significant (F_1,82_ 9.610, *p* < 0.01, partial eta-squared = 0.202). The overall duration of the hospital stay (in days) did not differ between groups (F_1,82_ = 1.532, ns). A general linear model analysis of the relationship between LAI use and CGI scores was also conducted and was not found to be significant (Figure 2).

## Discussion

Overall, our study gives a positive message for the continued use of LAI medications, with 70.6% of the dataset continuing on LAIs at 24 months post-discharge from an EIS for psychosis. However, consistent with the existing literature, this study does suggest that LAI discontinuation following discharge from an EIS is a potential contributing factor to decreases in treatment gains seen with EIS treatment for psychosis. We only saw trend level, not statistically significant associations between LAI use with ED visits, and this may be due to an overall low frequency of ED visits. However, maintenance of LAI use was significantly related to fewer inpatient admissions in our dataset. This is consistent with an outpatient study from the UK, which showed a reduction in inpatient stays (bed use) by an older cohort of patients with longer-standing psychotic disorders pre-post LAI initiation.^26^ While this study is not an economic analysis, these results (LAI continuation and its relation to hospital admission) counter a previous suggestion that LAI use may be too expensive,^12^ at least in the early phase psychosis population.

Our study shows that the decision to discontinue is more likely to be related to patient-driven reasons, which suggests that greater patient education on the long-term or ongoing benefits of LAI use may be a direction to improve compliance. Only two discontinuations were potentially driven by substitute decision makers, which could reflect the patient’s preference as well, but further education for this group could also potentially be helpful. Conversely, healthcare providers were often switching to clozapine, which would be appropriate for patients who were not experiencing a good treatment response on LAIs. Alternatively, providers switched due to patient preference for fewer side effects, which, given the impact of side effects on patient treatment adherence, would also be considered appropriate.^27^ While we do not have reasons for the LAI discontinuation for all patients, the use of clozapine or other treatments to reduce side effects supports the supposition that the trend line in Figure 2 for patients who discontinued LAI to be non-significantly higher than the LAI maintaining group is likely related to a positive bias in treatment efficacy and adherence among those who continued with non-LAI treatment.

The presence of individuals who stopped then restarted their LAI medication while still being treatment adherent was unexpected. It suggests that offering LAIs should not be discarded as an option even after an initial period of disuse or reluctance to use LAIs. It also may suggest that, despite stated negative views of LAIs by patients in this subset, past experience with LAIs demonstrated how efficacious the LAI use was. A piece of evidence from another research group supporting this view is recent work demonstrating that injectable formulations of paliperidone, while being less cost-effective than extended-release oral formulations, were associated with increased quality-adjusted life-years.^28^ Also consistent with this is a recent meta-analysis showing reduced all-cause and non-suicidal mortality in patients with schizophrenia who were on LAI as compared to those on oral formulations.^29^ Quality of life and return to LAI use were not a focus of our study, but could be an area for future investigation.

There are limitations to our study. This study was conducted in Canada, and many provincial health plans cover LAI medications for patients with a psychotic disorder. This lowers a barrier to continued LAI use that may not be the case in all parts of the world, but our study contributes to the evidence toward higher LAI use rates with greater financial coverage of the costs. Another limitation is that only 66% of the discharged patients (85 of 128) were able to be tracked for 2 years post-discharge. This is not unusual for studies in psychotic disorders to have a high loss to follow-up rate but which could lead to a positive bias in data. However, when the impact of these large losses on follow-up/high dropout rates on clinical outcomes has been examined in a meta-regression, the impact on study conclusions was not found to be significant.^30,31^ We could still be missing some of the most severely ill patients from this dataset. Concerningly, there were very few female subjects (and individuals who identified as women) discharged on LAIs from the EIS programs in this study. The reasons for this are unknown, but there was a significant gender imbalance in this dataset, with less than 10% of the sample identifying as women. There is no specific prohibition against females being prescribed LAI medication. However, this sex and gender bias is in keeping with a meta-analysis of the sex breakdown of clinical trials of LAIs, where only 34% of the participants were female.^32^ Clinician experience may also be part of this choice, with studies reporting that, overall, females experienced more side effects, particularly weight gain, with antipsychotic medications generally.^33^ It is possible that this undesirable side effect has resulted in more women discontinuing LAI use in the clinician experience, so over time, the practice may be to not prescribe to women. However, symptoms in women generally remit with lower doses of oral antipsychotic medications, which could be helpful in this regard.^33,34^ Antipsychotic medication response has also been reported to vary across reproductive phases and with contraception, and possibly vary across the menstrual cycle, leaving open the suitability of a standardized 30–90 day dosing of antipsychotic medication for women.^34^ This is an area that needs study, and these unanswered questions may be leading to under-prescribing of LAIs to women.^35^

This study adds to the evidence that for individuals with early phase psychosis, maintenance on an LAI is important to continued treatment success. A study on early psychosis patients examining predictors of readmission to inpatient care within 12 months showed oral antipsychotic use was a stronger predictor of hospital readmission than substance misuse, demonstrating the importance of continued promotion of LAI use.^20^ Despite the challenges in tracking the healthcare journey of patients with severe and persistent mental illness across health systems, this approach of following up after discharge can yield answers on the life course of psychotic disorders and response to treatment that can significantly inform treatment approaches. The inclusion of data from different health systems in different regions of one country is hoped to increase the generalizability of our findings, and we hope that this approach will give a broader understanding of the care pathways seen in various jurisdictions.

## Supplemental Material

sj-docx-1-tpp-10.1177_20451253251384509 – Supplemental material for Real-world examination of the rates of long-acting injectable attrition in a cohort of early psychosis patients after discharge from an early intervention service for psychosis Supplemental material, sj-docx-1-tpp-10.1177_20451253251384509 for Real-world examination of the rates of long-acting injectable attrition in a cohort of early psychosis patients after discharge from an early intervention service for psychosis by Candice E. Crocker, Thomas Hastings, Marc-Andre Roy, Nicola Otter and Philip G. Tibbo in Therapeutic Advances in Psychopharmacology

## Declarations

**Ethics approval and consent to participate:** This study received ethical approval from the three respective IRBs (Nova Scotia Health Research Ethics Board (REB file number 1028195), Halton Healthcare IRB (ref: LAI utilization study), and Comité d'éthique de la recherche sectorielle en santé mentale et dépendances du CIUSSS de la Capitale Nationale (Approval number 2024-2894, NSM) prior to study commencement. As this is an IRB-approved retrospective study, all patient information was de-identified, and a waiver for patient consent was approved by each IRB.

**Consent for publication:** Not applicable.

**Availability of data and materials:** Given the sensitive nature of this data (for privacy reasons), this dataset is not publicly available.
